# Open Surgical versus Minimal Invasive Necrosectomy of the Pancreas—A Retrospective Multicenter Analysis of the German Pancreatitis Study Group

**DOI:** 10.1371/journal.pone.0163651

**Published:** 2016-09-26

**Authors:** Sebastian Rasch, Veit Phillip, Stephanie Reichel, Bettina Rau, Christian Zapf, Jonas Rosendahl, Ulrich Halm, Markus Zachäus, Martin Müller, Alexander Kleger, Albrecht Neesse, Jochen Hampe, Mark Ellrichmann, Felix Rückert, Peter Strauß, Alexander Arlt, Volker Ellenrieder, Thomas M. Gress, Werner Hartwig, Ernst Klar, Joachim Mössner, Stefan Post, Roland M. Schmid, Thomas Seufferlein, Marco Siech, Jens Werner, Uwe Will, Hana Algül

**Affiliations:** 1 II. Medizinische Klinik und Poliklinik, Klinikum rechts der Isar, Technische Universität München, München, Germany; 2 Department for Gastroenterology, SRH Wald-Klinikum Gera, Gera, Germany; 3 Department of General, Thoracic, Vascular and Transplantation Surgery, University of Rostock, Rostock, Germany; 4 Division of Gastroenterology and Rheumatology, Department of Internal Medicine, Neurology and Dermatology, University of Leipzig, Leipzig, Germany; 5 Department of Internal Medicine II, HELIOS Park-Klinikum, Leipzig, Germany; 6 Department of Internal Medicine I, Ulm University, Ulm, Germany; 7 Department of Gastroenterology, Endocrinology, Infectiology and Metabolism, Philipps-University, Marburg, Germany; 8 Department of Gastroenterology and Gastrointestinal Oncology, University Medical Center Goettingen, Goettingen, Germany; 9 Department of Internal Medicine I, University Hospital Dresden, Dresden University of Technology, Dresden, Germany; 10 Department of Internal Medicine I, University Hospital Schleswig-Holstein, Kiel, Germany; 11 Department of Surgery, University Hospital Mannheim, University of Heidelberg, Heidelberg, Germany; 12 Department of General and Vascular Surgery, Ostalb-Klinikum Aalen, Aalen, Germany; 13 Department of General, Visceral, Transplantation, Vascular and Thoracic Surgery, Hospital of the University of Munich, München, Germany; Chinese University of Hong Kong Faculty of Medicine, HONG KONG

## Abstract

**Background:**

Necrotising pancreatitis, and particularly infected necrosis, are still associated with high morbidity and mortality. Since 2011, a step-up approach with lower morbidity rates compared to initial open necrosectomy has been established. However, mortality and complication rates of this complex treatment are hardly studied thereafter.

**Methods:**

The German Pancreatitis Study Group performed a multicenter, retrospective study including 220 patients with necrotising pancreatitis requiring intervention, treated at 10 hospitals in Germany between January 2008 and June 2014. Data were analysed for the primary endpoints "severe complications" and "mortality" as well as secondary endpoints including "length of hospital stay", "follow up", and predisposing or prognostic factors.

**Results:**

Of all patients 13.6% were treated primarily with surgery and 86.4% underwent a step-up approach. More men (71.8%) required intervention for necrotising pancreatitis. The most frequent etiology was biliary (41.4%) followed by alcohol (29.1%). Compared to open necrosectomy, the step-up approach was associated with a lower number of severe complications (primary composite endpoint including sepsis, persistent multiorgan dysfunction syndrome (MODS) and erosion bleeding: 44.7% vs. 73.3%), lower mortality (10.5% vs. 33.3%) and lower rates of diabetes mellitus type 3c (4.7% vs. 33.3%). Low hematocrit and low blood urea nitrogen at admission as well as a history of acute pancreatitis were prognostic for less complications in necrotising pancreatitis. A combination of drainage with endoscopic necrosectomy resulted in the lowest rate of severe complications.

**Conclusion:**

A step-up approach starting with minimal invasive drainage techniques and endoscopic necrosectomy results in a significant reduction of morbidity and mortality in necrotising pancreatitis compared to a primarily surgical intervention.

## Introduction

Acute pancreatitis (AP) is associated with an overall mortality of about 5% and due to an incidence between 30 and 45/100.000 person years a frequent and potentially fatal disease.[1–3] The mortality, however, depends on disease severity and may be as high as 20% in patients with severe and/or complicated pancreatitis. [2, 4, 5] Characteristics of moderate and severe pancreatitis are local or systemic complications. Local complications include acute peripancreatic fluid collections, pancreatic pseudocysts, acute necrotic collections and walled-off necrosis. On the other hand, systemic inflammatory response syndrome (SIRS) or sepsis, exacerbation of pre-existing co-morbidities and multi organ dysfunction syndrome (MODS) are typical systemic complications.[6] Necrotising pancreatitis evolves in about 30% of the patients with acute pancreatitis and is associated with a particularly poor outcome.[7, 8]

**Fig 1:**
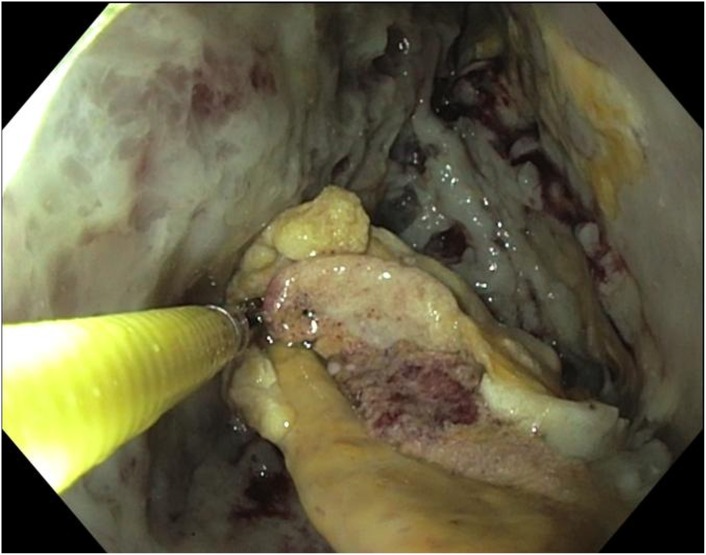
Endoscopic transgastric necrosectomy (ENS).

In the past nearly all patients with necrotising pancreatitis have been treated with open necrosectomy (ONS). ONS used to be performed early in the course of the inflammation, even in patients with sterile necrosis.[9] However, due to a high morbidity and the need of repetitive laparotomy, outcomes were unsatisfactory.[10, 11] During the last decade several studies showed better outcome for less invasive treatment approaches including transgastric and percutaneous drainage or endoscopic necrosectomy (ENS). Nowadays, it is generally accepted that intervention is only indicated if infected necrosis is suspected and that intervention should be delayed for at least 3–4 weeks after onset of pancreatitis if possible. The so called step-up approach consisting of conservative treatment followed by drainage and minimal invasive interventions results in a decrease in overall morbidity and defines the recommended standard care of therapy nowadays (Fig 1).[12–14]

However, the initial step-up approach of van Santvoort et al. did not include endoscopic drainage and necrosectomy. The majority of studies dealing with this technique so far were carried out by single specialized centers. Multicenter studies with a sufficient cohort size mainly focus on endpoints like MODS, resolution of necrosis and mortality. These studies lack important aspects such as number of interventions, length of hospital stay (LOS) and intensive care unit (ICU) stay and development of diabetes type 3c.[15]

This retrospective multicenter study aimed to compare ONS versus minimal invasive treatment of necrosis in patients with severe acute pancreatitis. The primary endpoint of this study is a composite endpoint of treatment associated morbidity (erosion bleeding, sepsis, persistent MODS) and mortality. Secondary endpoints are:

- treatment associated aspects like hospitalisation, number of interventions and infections
- complication rates of minimal invasive treatment options
- outcome after reconvalescence

We further evaluated possible prognostic or predictive factors for the development of complications in necrotising pancreatitis. The study was performed by the German pancreatitis study group (GPSG); by means of this group a large representative study population with patients from secondary and tertiary hospitals from all over Germany could be analysed.

## Patients and Methods

The study was approved by the ethics committee at Technische Universität München, Munich (project number 5726/16). In total, 7 tertiary referral centers and 3 secondary hospitals in Germany participated in this study. At the participating hospitals the local administrative databases were browsed for patients with pancreatitis by searching for the International Classification of Diseases (ICD)-10 code K85 (acute pancreatitis) and K86 (other diseases of pancreas). Patients with necrotising pancreatitis requiring treatment (percutaneous and/or transgastric/transduodenal drainage, surgical/percutaneous and/or endoscopic necrosectomy) in the late phase of pancreatitis (>10d after onset of symptoms) were included in the study. Necrotising pancreatitis was classified according to the revised Atlanta classification.[6] The patients were anonymised and details about history, diagnosis and treatment were collected in an Excel based data-base (Microsoft Office Excel 2007, Microsoft Cooperation, Redmond, USA). The study period was defined from January 2008 till June 2014 to avoid bias by changes in intensive care management, diagnostics, and treatment.

Sepsis was defined according to the European Society of Intensive Care Medicine (ESICM) criteria and MODS according to the modified Marshal scoring system.[6, 16] In accordance with the Atlanta classification persistent organ failure was defined as organ failure persisting for more than 48 hours. Interventions included percutaneous, endoscopic or surgical drainage and necrosectomy as well as any open surgery performed due to complications of necrotising pancreatitis.

### Statistics

Subgroups of the patients were formed for the comparison of specific parameters. Due to the retrospective design of the study, not all information could be obtained for each patient. Therefore, some of the subgroups included less than the total of 220 patients. In these cases the number of patients is mentioned separately.

Statistical analysis was performed with IBM SPSS Statistics 20 (SPSS Inc, Chicago, Illinois, USA) and if applicable as two sided test on a 5% level of significance. The level of significance was adjusted to 0.02 using the Benjamini-Hochberg procedure to control the false discovery rate by multiple testing due to the several primary and secondary endpoints. To compare qualitative parameters the chi-square test and in small samples (expected frequency of test variable less than 5) the Fisher's exact test were used. For the analysis of quantitative parameters the Mann-Whitney-U test and in case of a normal distribution the t-test were employed. A multivariate binary logistic regression model was used to analyse the effect of patient characteristics and a limited number of laboratory findings with high probability of impact as demonstrated by several other publications on the outcome of necrotising pancreatitis.

Descriptive data are presented as mean ± standard deviation (SD) for normally distributed parameters and median, range and interquartile range (IQR) for not normally distributed parameters. Risk ratios were displayed as odds ratio (OR) with 95% confidence interval (CI). Correlation was assessed with the Pearson and Spearman correlation coefficient.

## Results

### Patient's characteristics

At the 10 participating hospitals 220 patients were included (Table 1).

**Table 1:** Participating hospitals.

Hospital	Number of patients		
	Step-up group	ONS group	Total
Klinikum rechts der Isar, Technische Universität München	66	13	79
Department for Gastroenterology, SRH Wald-Klinikum Gera	35	1	36
Department of General, Thoracic, Vascular and Transplantation Surgery, University of Rostock	13	8	21
Division of Gastroenterology and Rheumatology, Department of Internal Medicine, Neurology and Dermatology, University of Leipzig	19	1	20
Klinik für innere Medizin II, HELIOS Park-Klinikum Leipzig	19	0	19
Department of Internal Medicine I, Ulm University	17	1	18
Department of Gastroenterology, Endocrinology, Infectiology and Metabolism, Philipps-University, Marburg	10	0	10
Department of Internal Medicine I, University Hospital Schleswig-Holstein, Kiel	10	0	10
Department of Surgery, University Hospital Mannheim, University of Heidelberg	0	6	6
Department of General and Vascular Surgery, Ostalb-Klinikum Aalen	1	0	1
**∑**	**190**	**30**	**220**

Primary open surgical necrosectomy was performed on 30/220 (13.6%) patients while the remaining 190/220 (86.4%) were treated according to a step-up approach. However, 36/190 (18.9%) patients within the step-up group needed an open surgical intervention later in the course of the disease. Detailed patients characteristics are listed in Table 2.

**Table 2:** Patient characteristics.

	All patients	Step-up	ONS
**n**	220	190	30
**♂: ♀**	2.6:1	2.7:1	2.3:1
		p = 0.888	
**median age**	58	58	55
	(range 18–88; IQR 46–70)	(range 22–88; IQR 47–75)	(range 18–82; IQR 43–64)
		p = 0.142	
**Genesis**	41.4% (91/220)	Biliary	
	29.1% (64/220)	Alcoholic	
	13.6% (30/220)	Iatrogen	
	2.7% (6/220)	Drug induced	
	1.8% (4/220)	Hypertriglyceridemia	
**status post AP**	43.2%	49.5%	3.3%
	(95/220)	(94/190)	(1/30)
		p<0.001	
**status post necrotising pancreatitis**	3.2%	3.7%	0.0%
	(7/220)	(7/190)	(0/30)
**history of CP**	17.3%	18.4%	10.0%
	(38/220)	(35/190)	(3/30)
**median number of interventions**	4	3.3	5.5
	(range 1–78; IQR 2–8)	(range 1–78; IQR 2–7)	(range 1–35; IQR 3–15)
		p = 0.038	

ONS, open necrosectomy; AP, acute pancreatitis; CP, chronic pancreatitis

Before intervention criteria for SIRS, mean arterial pressure (MAP) and time between the first intervention and onset of symptoms were not significantly different in both groups.

### Treatment

With regard to the composite endpoint, 44.7% (85/190) of the patients in the step-up group and 73.3% (25/30) in the surgical group suffered at least one severe (sepsis, persistent MODS or erosion bleeding) complication (p<0.001).

**Fig 2:**
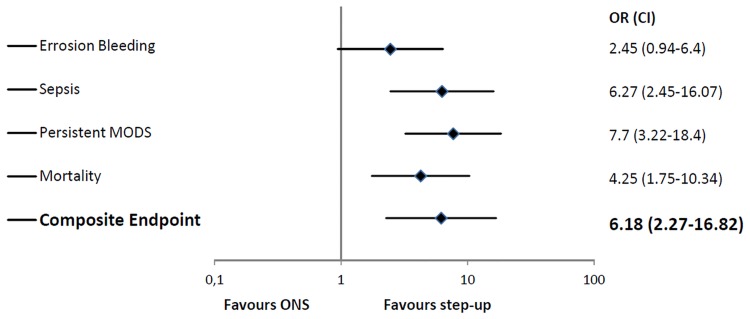
Severe complications and mortality (open necrosectomy: ONS, odds ratio: OR, confidence interval: CI).

Overall 13.6% (30/220) of the patients died in hospital or within 4 weeks after discharge. Consistently with severe complications, the mortality rate was significantly lower in the step-up group when compared to initially surgically treated patients (10.5% (20/190) versus 33.3% (10/30); p = 0.002) (Fig 2).

The overall median length of hospital stay was 43 days (range 16–367; IQR 24–86). The difference in the median length of hospital stay between the step-up and the surgical group was not statistically significant (median 42; range 16–367; IQR 24–80 versus 74; range 21–239; IQR 31–108; p = 0.048).

The median length of stay on ICU was 21 days with a wide range from 1 to 209 days (IQR 5–45). There was no statistical significant difference between the two subgroups (median 19; range 1–209; IQR 5–36 versus median = 28; range 1–100; IQR 12–66; p = 0.124).

Ninety percent (197/220) of all patients were treated either with a percutaneous drainage, a transgastric drainage or both. Without another intervention 50.8% (100/197) of these patients recovered and 49.2% (97/197) underwent minimally invasive necrosectomy.

The median number of interventions performed per patient was 4 (range 0–78; IQR 2–8) and did not differ significantly between the two subgroups. In the step-up group 18.9% (36/190) of the patients required an open surgical intervention after drainage placement or minimal invasive necrosectomy. In 49.1% (108/220) of the patients three or more groups of antibiotics were administered during the course of the disease. There was no statistical significant difference in the number of different antibiotics between the two groups (p = 0.093).

Confirmed or suspected infection of pancreatic necrosis was the most frequent indication for intervention. In 72.7% (160/220) of the patients, a pathogen was identified. There was no statistical significant difference in the frequency of infected necrosis between the subgroups (71.1% (135/190) in the step-up group versus 83.3% (25/30) in the surgical group p = 0.191).

On average 3.3 (SD ±2.35) pathogens were isolated in patients with confirmed infected necrosis. Most frequently Enterococcus faecium was cultured followed by Candida albicans, Enterococcus faecalis and Eschericha coli (Table 3).

**Table 3:** Cultured pathogens in infected pancreatic necrosis.

cultured pathogen	n patients
Enterococcus faecium	78
Candida albicans	58
Enterococcus faecalis	41
Eschericha coli	40
Staphylococcus epidermidis	32
Candida glabrata	26
Klebsiella pneumoniae	23
Staphylococcus aureus	21
Bacteroides	18
Streptococcus anginosus	17
Pseudomonas aeruginosa	16
Coagulase negative staphylococci	15
Stenotrophomonas maltophilia	14
Enterobacter cloacae	13
Staphylococcus haemolyticus	11

In total, 201/220 (91.4%) patients of both groups received either a percutaneous or a transgastric/transduodenal drainage or both. Severe complications (erosion bleeding, sepsis, persisting MODS) and mortality were significantly less frequent in patients treated by transgastric drainage when compared to patients treated with percutaneous drainage (severe complications and mortality 32.1% (27/84) versus 58.1% (68/117), p<0.001, OR 0.34, CI 0.19–0.61). There was no statistical significant difference in the rate of severe complications or mortality comparing the combination of percutaneous and transgastric drainages to one of these drainages alone (47.6% (69/145) versus 46.3% (25/54), p = 0.875). There was a significantly lower rate of severe complications and mortality in patients treated by ENS following a drainage placement compared to patients who received no ENS after drainage placement (35.1% (34/97) versus 58.7% (61/104), p = 0.001, OR 0.38, CI 0.21–0.67). There was no significant difference in the distribution of age, heart rate, respiratory rate, temperature, white blood cell count, hematocrit, blood urea nitrogen and history of AP between the comparison groups in this paragraph. Patients of the step-up approach group who required secondary surgery had a significantly higher rate of severe complications and mortality than those without secondary surgery (40.3% (62/154) versus 69.4% (25/36), p = 0.001).

### Follow up

Only 19 of 220 (8.6%) patients developed new onset diabetes after necrotising pancreatitis. In the step-up group diabetes type 3c emerged in 4.7% (9/190) and in the surgical group in 33.3% (10/30) of the patients (p<0.001). In total, 21.8% (48/220) of the patients had persistent dysfunction of another organ in the follow up period. This finding was significantly less frequent in the step-up group than in the surgical group (16.8% (32/190) versus 53.3% (16/30); p<0.001).

**Fig 3:**
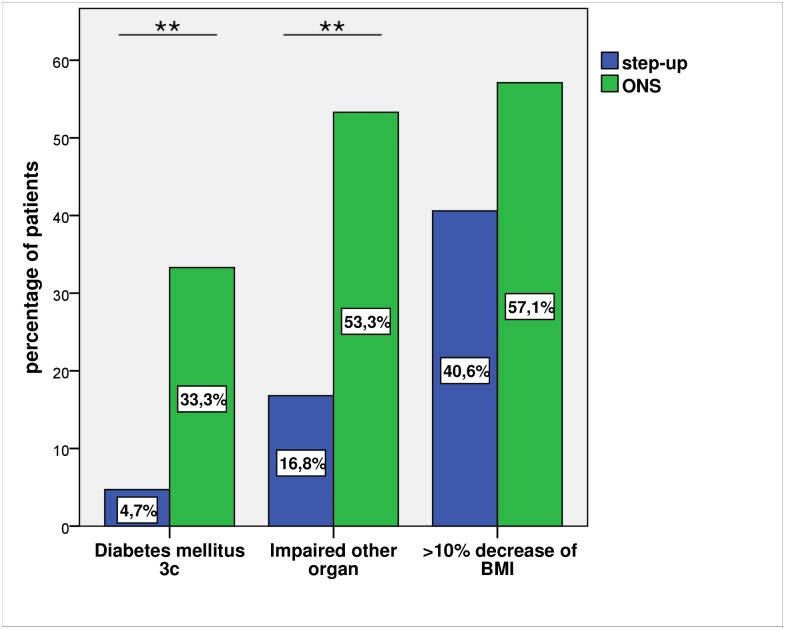
Follow up (** p<0.01); Impaired other organ: irreversible damage of other organs like gastrointestinal passage, impairment of renal or liver function.

Weight loss of more than 10% of the body weight as a surrogate parameter of decrease in the patients general condition during the hospital stay occurred in 42.1% (32/76) without a statistical difference between the subgroups of the step-up and the surgical group, respectively (40.6% (28/69) versus 57.1% (4/7); p = 0.446) (Fig 3).

### Risk factors or prognostic factors for the development of complications

A white blood cell (WBC) count > 20 G/l, C-reactive protein (CRP) > 15 mg/dl, hematocrit > 44% and blood urea nitrogen (BUN) > 25 mg/dl are established prognostic parameters in early acute pancreatitis.[5] Multivariate logistic regression analysis was performed to test the influence of WBC, CRP, hematocrit, BUN at admission and history of AP as well as age, gender and BMI on the development of severe complications and mortality. This analysis showed that hematocrit, BUN and history of AP are independent prognostic parameters for the development of severe complications or mortality during the course of necrotising pancreatitis (hematocrit >44%: OR = 4.87, CI = 1.70–13.89, p = 0.003; BUN>25 mg/dl: OR = 3.39, CI = 1.32–8.73, p = 0.011; history of AP: OR = 0.29, CI = 0.13–0.65, p = 0.002). The other variables of the regression model had no significant association with the composite primary endpoint.

## Discussion

This retrospective, multicentre study is one of the first real-life studies evaluating the step-up approach and open surgical necrosectomy in daily clinical practice. We observed that patients treated according to a step-up approach with minimal invasive drainage techniques followed by endoscopic necrosectomy showed a significant lower morbidity and mortality rate in necrotising pancreatitis compared to a primary surgical intervention. Further, our data add new aspects to this widely used approach in the management of necrotising pancreatitis.

While previous studies failed to demonstrate a difference in mortality between the surgical and step-up group, our data clearly reveal a significant reduction in mortality. Particularly the mortality rate of 10.5% in the step-up group is markedly lower than the earlier described 18–27%. The overall mortality was well in line with several studies (13.6% versus 15% mortality for necrotising pancreatitis in previous studies). The mortality rate of about 30% in patients treated by primary open necrosectomy is also in line with the published data.[12, 14, 17, 18] One possible explanation for the comparatively low mortality rate in the step-up group might be the continuous replacement of minimal retroperitoneal necrosectomy by modern endoscopic drainage techniques. And yet, studies showing superiority of endoscopic over minimal invasive retroperitoneal surgical necrosectomy are missing. Also a delayed time of intervention and advanced intensive care treatment options might contribute to these findings.[19, 20]

The initial study of van Santvoort et al. along with this study, reveal a significant reduction in the primary composite endpoint. MODS was included in both composite endpoints and is considered as a major predictor of mortality in acute pancreatitis. This has been addressed in numerous studies.[21] In contrast to the van Santvoort study we assessed persistent rather than new onset MODS. However, in both studies a significant reduction in MODS has been described. Likewise, sepsis as another severe complication occurred less in the step-up group, whereas there is no difference concerning intra-abdominal bleeding in either study.[14]

Duration of hospitalisation has not only a relevant impact on the patient's quality of life, but has also a significant implication on health-economic issues. Our study reveals a trend towards a shorter hospital stay of and less interventions in patients with minimal invasive therapy. More than the number of interventions the duration of ICU stay is cost-intensive. The median time on ICU is comparable in both groups. Of note, after major pancreatic surgery, patients are routinely monitored on ICU. With a wide range for the time of both hospitalisation and ICU stay in the step-up group the individual course of the disease is heterogeneous.

Patients in the step-up group had less long term complications such as diabetes mellitus type 3c after reconvalescence. This is another important factor for quality of life and subsequent health care costs.[22, 23] However this observation needs to be validated by a study with a longer, standardised follow up of patients with necrotising pancreatitis.

Almost one fifth of the patients receiving minimally invasive therapy also required a surgical intervention during the course of the disease. These patients had a high morbidity and mortality rate. So despite the decreasing role of open necrosectomy in the initial treatment of necrotising pancreatitis surgery maintains an important part in the management of complications (e.g. abdominal compartment syndrome or perforation). This highlights the complexity in the treatment of severe acute pancreatitis and more tailored studies are required to address this topic.

Furthermore this study shows that a step-up approach was an accepted and successfully applied therapeutic concept even before the study of van Santvoort et al.

Besides percutaneous drainage placement and necrosectomy, endoscopy provides new treatment options and more than half of the patients in this study were treated endoscopically with even less severe complications and lower mortality. Thus, in selected patients transgastric drainage and endoscopic necrosectomy seem to be safe and effective, although these findings might be biased by a non randomized selection of patients for endoscopic intervention. This has also been suggested by recent studies.[18, 24, 25] In contrast to other studies patients did not benefit from combined percutaneous and transgastric drainage.[26] Drainage combined with minimal invasive necrosectomy was associated with the lowest complication rate in this study. In line with the literature an early minimal invasive necrosectomy of walled off necrosis might turn out as preferred treatment in patients with necrotising pancreatitis and an indication for intervention.[27] Still, the choice of interventions should also depend on local skill, expertise and localization of necrosis, which are important factors for post interventional complications.[10, 11]

One important complication of necrotic pancreatic tissue is superinfection. Infection of necrosis was very common in our patient population and equally distributed between the subgroups. As it was the indication for intervention in many cases and not the consequence of the interventions, infection of necrosis was not considered as a treatment related complication. Bacteria of the gastrointestinal tract are reported as typical pathogens in infected pancreatic necrosis. A relatively high number of Enterococcus species was registered in our patient population.[28, 29]

The prognostic role of hematocrit and BUN for the prediction of severe acute pancreatitis has been well established.[5, 30] Being an important marker for inflammation, CRP serum levels higher than 15 mg/dl turned out to be a useful prognostic tool to predict severity of pancreatitis 48h after admission.[31, 32] In this study there was no association of CRP serum levels with complications in necrotising pancreatitis. To conclude hematocrit and BUN can not only discriminate between mild and severe pancreatitis but also seem to be predictive for complications in severe necrotising pancreatitis. Interestingly, a history of acute pancreatitis is associated with a decreased risk of severe complications. However this observation might be due to the fact that these patients have a higher incidence of chronic pancreatitis and therefore acute attacks are less complicated.

This study has several limitations. With its retrospective design no detailed data on comorbidities, Ranson criteria or BISAP score could be obtained.[33] And yet, both groups did not differ in several key values such as SIRS criteria, WBC, BUN and age.[34, 35] Medical conditions of the patients included were comparable. Additional to the retrospective design of the study, there are considerable more patients treated according to a step-up approach than by primarily surgical necrosectomy. This asymmetrical distribution of therapeutic approaches reflects the factual decrease of primary surgical treatment.

## Conclusion

Minimal invasive treatment of necrotising pancreatitis is associated with less complications, lower mortality and better outcome. According to our data, patients treated with transgastric drainages in combination with endoscopic necrosectomy had the lowest morbidity and mortality.
